# Trends of multimorbidity in 15 European countries: a population-based study in community-dwelling adults aged 50 and over

**DOI:** 10.1186/s12889-020-10084-x

**Published:** 2021-01-07

**Authors:** Dyego L. B. Souza, Albert Oliveras-Fabregas, Eduard Minobes-Molina, Marianna de Camargo Cancela, Paola Galbany-Estragués, Javier Jerez-Roig

**Affiliations:** 1 Department of Collective Health, Postgraduate Programme in Collective Health, Federal University of Rio Grande do Norte, Natal, Brazil; 2 Research group on Methodology, Methods, Models and Outcomes of Health and Social Sciences (M3O). Faculty of Health Sciences and Welfare. Centre for Health and Social Care Research (CESS), University of Vic-Central University of Catalonia (UVic-UCC), C. Sagrada Família, 7, 08500 Vic, Spain; 3 Physical Activity, Sport and Health Research Group. Faculty of Psychology, Education and Sport Sciences Blanquerna, Universitat Ramon Llull, Barcelona, Spain; 4 Division of Population Research, Brazilian National Cancer Institute, Rio de Janeiro, Brazil

**Keywords:** Prevalence, Older adults, Non-communicable diseases, Europe, SHARE

## Abstract

**Background:**

The objective of this work was to analyse the prevalence trends of multimorbidity among European community-dwelling adults.

**Methods:**

A temporal series study based on waves 1, 2, 4, 5, 6 and 7 of the Survey of Health, Ageing and Retirement in Europe (SHARE) was conducted, and community-dwelling participants aged 50+ (*n* = 274,614) from 15 European countries were selected for the period 2004–2017. Prevalence, adjusted by age, Average Annual Percentage Change (APC) and 95% confidence interval (95% CI) were all calculated. Trend analyses were realised by period, age groups and groups of diseases.

**Results:**

The results showed a large variability in the prevalence of multimorbidity in adults aged 50 and over among European countries. Increase in the prevalence of multimorbidity in the countries of central Europe (Austria, Belgium, Czech Republic, France, Germany and Switzerland) and Spain in both sexes, and in the Netherlands among men. Stability was observed in northern and eastern European countries. Musculoskeletal and neurodegenerative groups showed more significant changes in the trend analyses.

**Conclusions:**

This information can be useful for policy makers when planning health promotion and prevention policies addressing modifiable risk factors in health.

## Background

Overall life expectancy and healthy life years have increased worldwide, but quality of life and functional capacity have worsened due to non-communicable diseases strongly related to ageing [1]. These changes in population ageing contribute to a higher prevalence of multimorbidity, defined as the presence of two or more chronic conditions in the same individual [2].

Multimorbidity has become a major public health concern, challenging patients, health care providers, and health care systems. Multiple chronic conditions have a strong impact on the affected population, including poor health outcomes, low quality of life, high health care utilisation and increase in expenses [3, 4]. The need to prevent the consequences of these diseases has become important for public health actions. Therefore, it is necessary to understand the prevalence trends of multimorbidity in populations and their impact over time.

Although this is a relevant and clinically important topic, there is a lack of studies on trends in the prevalence of chronic diseases. Scarce research investigating trends in multimorbidity points towards increases in multimorbidity prevalence over time [5, 6]: the prevalence of chronic diseases and multimorbidity increased over the period 2001–2011 [6] and this trend is expected to continue in the context of population ageing.

In 2011, one study predicted that in 2015 multimorbidity among patients over 65 years of age would be over 30% [7]. This research took into account three types of chronic diseases: diabetes, pulmonary and cardiovascular diseases, and stated that their results may show an underestimation of the real situation, because other chronic multimorbidity diseases, like arthrosis, were left out of consideration [7]. Another aspect that needs to be studied further is the association of multimorbidity with gender, since women have been associated with multimorbidity in some countries [8, 9].

For all these reasons, it is necessary to carry out an updated analysis of the trends of multimorbidity in Europe, studying the issue by age groups and including more chronic diseases: cardiometabolic, musculoskeletal, respiratory, cancer and neurodegenerative disorders.

Therefore, the present study sought to analyse the prevalence trends of multimorbidity among community-dwelling adults aged 50 and over in 15 European countries.

## Methods

An ecological study with temporal series population-based analysis was realised using data from waves 1, 2, 4, 5, 6 and 7 of the Survey of Health, Ageing and Retirement in Europe (SHARE) project (www.share-project.org). Wave 3 did not collect information about non-communicable diseases. SHARE is a longitudinal, multidisciplinary, cross-country, research project conducted in Europe about ageing. It was approved by the Ethics Committee of the Max Planck Society and the countries’ implementations by the respective ethics committees or institutional review boards [10]. The SHARE Project respects the Declaration of Helsinki in terms of anonymity of the participants; informed consent for study participation was obtained from all participants and/or their legal guardians. Further details about sampling procedures, data collection and other methodology aspects can be found elsewhere [10–12].

Data from the following 15 European countries were included: Austria, Belgium, Czech Republic, Denmark, Estonia, France, Germany, Netherlands, Italy, Poland, Portugal, Spain, Sweden, Slovenia and Switzerland. All countries obtained probabilistic samples, with slight sampling differences [11]. Adults permanently living in nursing homes were excluded. The prevalence for year 2009 was estimated by lineal interpolation (Wave 3) [13]. The prevalence for year 2013 was also estimated for Poland and Portugal, because these countries did not participate in wave 5. Countries with data of less than four consecutive waves or allowing interpolation estimation of one wave were excluded.

Dependent variable multimorbidity was defined as the coexistence of two or more chronic conditions [14]. Eleven non-communicable diseases were included and grouped into five diseases groups: cardiometabolic disorders (high blood pressure or hypertension, diabetes or high blood sugar, heart attack - including myocardial infarction or coronary thrombosis or any other heart problem including congestive heart failure, stroke and high cholesterol); musculoskeletal disorders (osteoarthritis or rheumatoid arthritis and hip fracture or femoral fracture); respiratory disorder (chronic lung disease); cancer (malignant tumor - excluding minor skin cancers) and neurodegenerative disorders (Parkinson’s disease, Alzheimer’s disease, dementia or senility). Information about non-communicable diseases was obtained through self-report. Since the information about Alzheimer’s disease was not asked in wave 1, we input the prevalence estimate of Alzheimer’s disease of wave 2.

The analyses of the prevalence for each disease group were realised, stratified by gender and country. Prevalence trends were calculated by period, age groups (50–59, 60–69, 70–79 and 80 plus) and disease group. Prevalence by period and disease group was adjusted by five-year age groups (50–54, 55–59, 60–64, 65–69, 70–74, 75–79 and 80 plus).

Statistical trend analyses were carried out using Stata software, version 14.0, and the Joinpoint Regression Program software version 4.7.0.0. Calibrated individual weights, provided in the SHARE dataset, were applied to the analyses. The estimated annual percent change (Average Annual Percent Change - AAPC) and corresponding 95% confidence intervals (CI) were calculated [13]. Prevalence was used as the dependent variable and the year as the independent variable. In all analyses, a *p* value lower than 0.05 was considered statistically significant.

## Results

**Fig. 1:**
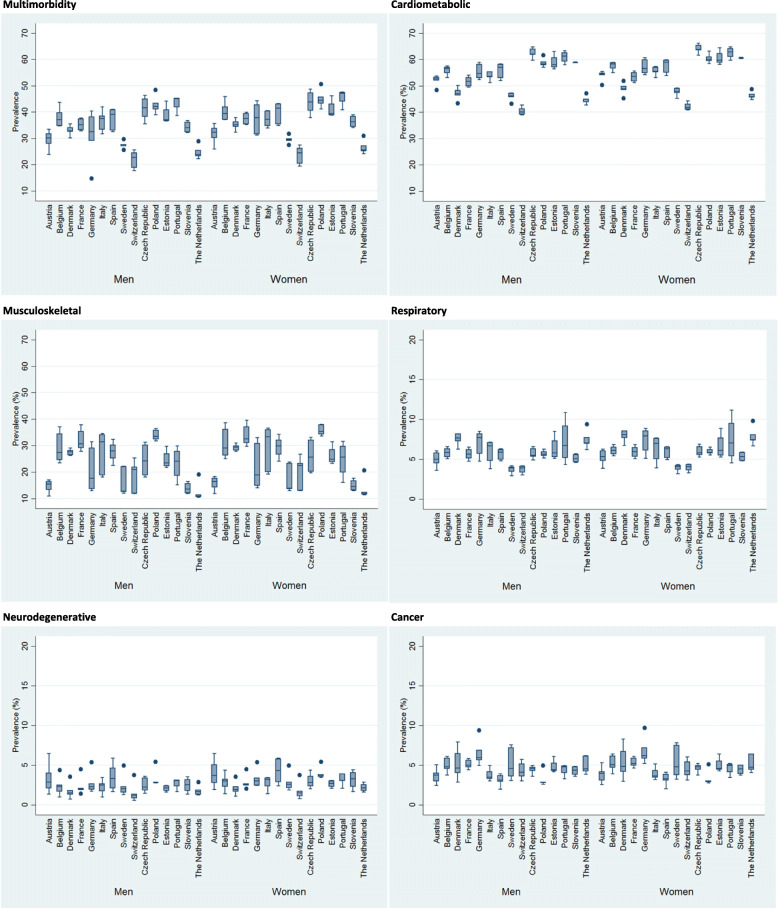
Variability in prevalence of multimorbidity between the 7 waves (2004–2017) studied by groups of disease types in people aged 50 and over in 15 European countries

The sample consisted of 274,614 individuals. Figure 1 shows variability in prevalence of multimorbidity between the 7 waves (2004–2017) studied by groups of disease types. The prevalence was higher in Portugal, Poland, Czech Republic and Estonia, and lower in Switzerland, Sweden and Netherlands. Cardiometabolic and musculoskeletal diseases were more prevalent while cancer and neurodegenerative diseases were less prevalent.

**Fig. 2:**
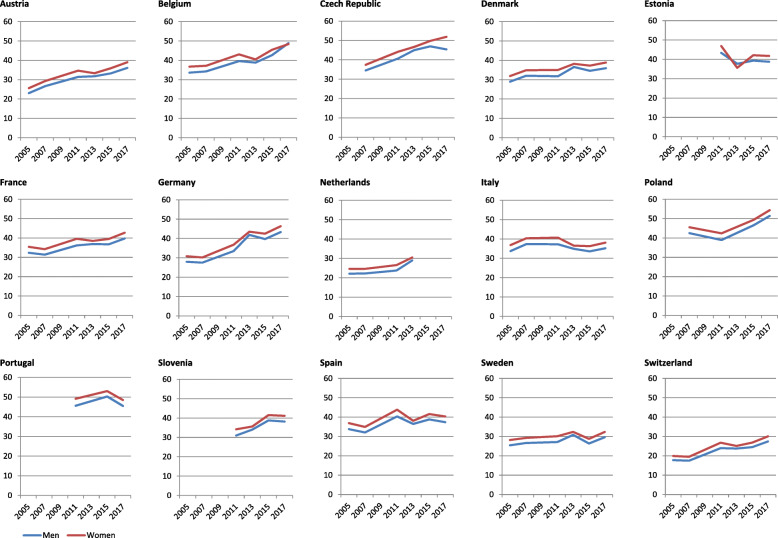
Prevalence trends of multimorbidity in 15 European countries in community-dwelling men and women aged 50 and over

The trend analyses by period show an increase of multimorbidity prevalence in both genders in Austria (APC = 2.3% in men and APC = 2.2% in women), Belgium (APC = 1.7 in men and APC = 1.5% in women), Czech Republic (APC = 2.7% in men and APC = 2.6% in women), France (APC = 1.0% in men and APC = 0.9% in women), Germany (APC = 6.9% in men and APC = 3.2% in women), Spain (APC = 2.0% in men and APC = 1.9% in women) and Switzerland (APC = 2.7% in men and APC = 2.6% in women). In the Netherlands, the increase was observed in men, APC = 2.9% (see Fig. 2 and Table 1). For the rest of the countries, the prevalence remained relatively stable during the period.

**Table 1:** Prevalence of multimorbidity by country, age group and gender among people aged 50 and over

	Years	Period	95% CI		50–59	95% CI		60–69	95% CI		70–79	95% CI		80+	95% CI	
		AAPC	Lower	Upper	AAPC	Lower	Upper	AAPC	Lower	Upper	AAPC	Lower	Upper	AAPC	Lower	Upper
**Men**																
Austria	2004–2017	2.3^a^	0.9	3.7	2.4^a^	0.7	4.2	1.9^a^	0.3	3.6	2.2^a^	1.8	2.6	3.6^a^	0.8	6.6
Belgium	2004–2017	1.7^a^	0.4	2.9	2.7^a^	0.1	5.3	2.2^a^	1.7	2.6	1.8^a^	0.5	3.1	0.5	−0.6	1.6
Czech Republic	2007–2017	2.7^a^	2.2	3.3	4.6^a^	2.7	6.6	1.4^a^	0.1	2.8	3.3^a^	2.2	4.5	2.9^a^	1.8	4.0
Denmark	2004–2017	0.9	−0.0	1.8	0.9	−0.9	2.7	1.2	−0.6	3.1	1.4^a^	0.2	2.7	2.2	−1.0	5.4
Estonia	2011–2017	−2.6	−9.1	4.3	−4.8^a^	−8.0	−1.4	− 1.7	− 14.3	12.7	−3.5	− 10.0	3.5	− 1.3	−9.7	8.0
France	2004–2017	1.0^a^	0.1	1.9	0.6	−2.7	4.1	−0.3	−1.3	0.7	1.5	− 0.7	3.8	0.9	−0.8	2.7
Germany	2004–2017	6.9^a^	1.8	12.3	5.0^a^	0.4	9.9	3.0^a^	0.3	5.9	2.6^a^	1.3	3.9	0.5	−0.1	1.1
Netherlands	2004–2017	2.9^a^	0.2	5.8	4,8	−3,8	14,3	0,4	−1,1	1,9	3,5	−1,5	8,8	4,3	−0,4	9,3
Italy	2004–2017	−1.6	−3.3	0.1	−4.4^a^	−6.4	−2.3	− 3.2^a^	−4.9	− 1.4	− 0.2	− 1.4	1.1	2.4^a^	1.5	3.3
Poland	2007–2017	1.2	−1.0	3.4	−0.5	−4,1	3.2	−0.0	− 1.4	1.4	2.3^a^	0.3	4.3	4.4^a^	0.2	8.9
Portugal	2011–2017	−2.3	−8.0	3.8	− 2.1	−16.9	15.2	−4.1	−11.4	3.7	1.0	−5.3	7.7	−0.0	−16.2	19.2
Slovenia	2011–2017	2.1	−2.2	6.6	0.9	−4.1	6.1	3.8	−7.9	17.0	3.5	−1.5	8.9	5.6	−2.8	14.7
Spain	2004–2017	2.0^a^	0.7	3.4	5.3^a^	2.2	8.6	1.8^a^	0.0	3.7	2.7^a^	0.9	4.5	3.1^a^	1.6	4.6
Sweden	2004–2017	−0.0	−1.2	1.1	1.3	−2.6	5.4	−1.0	−3.1	1.2	−0.4	−1.4	0.6	−0.2	−2.6	2.2
Switzerland	2004–2017	2.7^a^	0.9	4.6	1.6	−0.2	3.4	1.3	−1.2	4.0	2.9^a^	1.1	4.3	5.4^a^	3.2	7.7
**Women**																
Austria	2004–2017	2.2^a^	0.9	3.5	−1.3	−6.5	4.3	0.3	−1.2	1.9	3.3^a^	1.5	5.1	4.0^a^	2.2	5.8
Belgium	2004–2017	1.5^a^	0.4	2.7	1.7	−1.4	4.9	0.9	−0.5	2.3	1.0^a^	0.2	1.8	0.7	−0.2	1.7
Czech Republic	2007–2017	2.6^a^	2.1	3.0	4.0^a^	2.3	5.7	1.4^a^	0.3	2.5	2.1^a^	1.4	2.8	2.0	−0.1	4.1
Denmark	2004–2017	0.8	−0.1	1.7	−1.3^a^	−2.1	−0.4	0.6	−1.3	2.4	0.3	−1.0	1.5	0.8	−1.1	2.8
Estonia	2011–2017	−2.4	−8.4	3.9	−4.2	− 10.2	2.3	−3.2	− 7.0	0.8	−1.9	−7.6	4.2	−0.6	− 4.2	3.2
France	2004–2017	0.9^a^	0.2	1.7	−1.8	−4.2	0.7	1.5^a^	0.9	2.0	0.8	−0.5	2.1	1.6^a^	0.4	2.9
Germany	2004–2017	3.2^a^	2.0	4.4	5.0^a^	3.1	7.0	2.3^a^	1.3	3.3	3.5^a^	1.9	5.1	2.1^a^	1.4	2.8
Netherlands	2004–2017	2.7	−0.0	5.6	0,1	−2,5	2,8	1,2	−3,3	6	3,1	−1	7,3	4,2	−5,4	14,8
Italy	2004–2017	− 1.2	−2.7	0.3	− 5.0^a^	−7.1	− 3.0	− 2.5^a^	−4.6	−0.3	− 0.9	− 2.4	0.6	0.9	− 0.8	2.5
Poland	2007–2017	1.1	− 1.1	3.3	1.2	−3.6	6.2	0.1	−1.6	1.8	0.5	−0.7	1.8	3.0	−0.5	6.7
Portugal	2011–2017	−2.1	−7.6	3.8	−3.3	−8.0	1.7	−4.0	−16.9	10.9	−1.6	− 11.9	9.8	1.0	−2.9	5.1
Slovenia	2011–2017	2.1	−2.0	6.4	−4.5^a^	−7,8	−1,1	−0,5	−6,6	6	3,6	−3,9	11,8	4,9	−2,6	13
Spain	2004–2017	1.9^a^	0.7	3.1	−0.2	−3.0	2.7	−0.4	−1.9	1.1	1.2^a^	0.4	2.0	3.1^a^	1.4	4.9
Sweden	2004–2017	−0.1	−1.2	1.1	−1.1	−4.1	2.0	0.2	−1.4	1.8	0.2	−1.1	1.4	−0.6	−2.6	1.4
Switzerland	2004–2017	2.6^a^	1.0	4.4	3.9^a^	0.0	8.0	4.1^a^	0.8	7.5	0.6	−0.6	1.9	3.5	−0.5	7.5

^a^Statistically significant (*p* < 0.05)

**Fig. 3:**
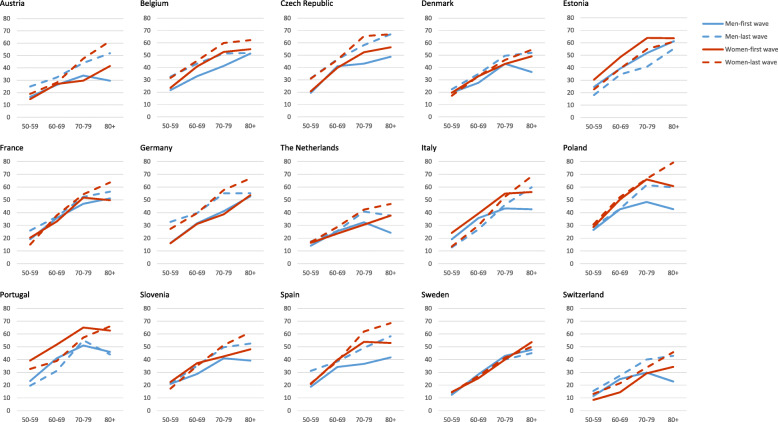
Prevalence of multimorbidity by country, age group and gender in the first and last SHARE wave among people aged 50 and over

When analysing the age groups, it was possible to find differences by country and gender, and the increase of multimorbidity prevalence with age was observed (Table 1 and Fig. 3). Austria showed a significant increase in all age groups or women and all age groups in men except for those aged 70 and over. The Czech Republic presented a significant increase in all age groups in both sexes, except women aged over 80. In Belgium, an increasing trend was registered in the age groups between 50 and 79 years in men and 50–59 and 70–79 in women. In Germany, there was an increase in all age groups in women and in 50–79 years in men, with a more evident increase in the younger groups. A significant increase in Denmark in the 70–79 age group in men and decrease in women only in the 50–59 age group were verified. In Spain, there was an increase in men and women aged 70 or over, and in Poland an increase in men aged 70 and over. In Italy, an increase in men aged 80 and over, along with a significant reduction in the younger groups in both women and men was observed. In Switzerland, there was an increase in men aged 70 or over, while younger women presented an increase. In France, only an increase in women was observed.

Cardiometabolic, musculoskeletal and respiratory groups showed more significant changes in the trend analyses (Table 2). Cardiometabolic diseases increased in Austria, Germany and Spain, and respiratory diseases increased in Austria, Belgium, Germany, Portugal, Sweden and Switzerland. In Italy, a reduction in respiratory and musculoskeletal diseases was observed, and in Portugal a reduction in cardiometabolic and musculoskeletal diseases. The prevalence of musculoskeletal diseases increased in northern and central countries (Belgium, Czech Republic, France, Germany, Sweden and Switzerland), and neurodegenerative diseases increased in Estonia and Slovenia, in both men and women, while in Spain and Italy they increased in women. For cancer, no changes were identified changes.

**Table 2:** Multimorbidity prevalence according to disease group by country and gender among people aged 50 and over

	Years	CM	95% CI		RE	95% CI		MS	95% CI		ND	95% CI		CA	95% CI	
		AAPC	Lower	Upper	AAPC	Lower	Upper	AAPC	Lower	Upper	AAPC	Lower	Upper	AAPC	Lower	Upper
**Men**																
Austria	2004–2017	0.6^a^	0.0	1.2	3.8^a^	1.7	5.9	1.3	−2.4	5.2	6.5	−5.0	19.4	0.2	−5.9	6.7
Belgium	2004–2017	−2.0	−0.9	0.5	2.0^a^	0.8	3.2	3.7^a^	2.0	5.5	5.1	−5.4	16.8	−0.3	−4.6	4.2
Czech Republic	2007–2017	0.6	−0.1	1.3	1.1	−2.7	5.1	6.3^a^	1.6	11.3	3.7	−8.4	17.5	−0.6	−4.5	3.6
Denmark	2004–2017	0.6	−0.4	1.5	1.1	−0.9	3.2	−0.5	−1.3	0.4	2.1	−9.5	15.0	−4.6	−11.3	2.5
Estonia	2011–2017	−1.7	−3.8	0.4	−6.8	−20.4	9.1	− 2.9	−15.1	11.1	7.1^a^	2.3	12.1	−4.4	− 18.0	11.4
France	2004–2017	−0.5	−1.2	0.2	0.6	−2.2	3.5	2.3^a^	0.8	3.8	0.1	−8.9	10.1	−0.7	−3.0	1.8
Germany	2004–2017	0.9^a^	0.1	1.6	4.1^a^	0.7	7.7	8.8^a^	6.0	11.7	0.7	−8.7	11.1	2.0	−2.9	7.1
Netherlands	2004–2017	0.5	−1.5	2.5	4.1	−1.0	9.5	5.0	−6.7	18.1	1.0	−16.6	22.4	0.9	−10.9	14.4
Italy	2004–2017	−0.3	−1.0	0.3	−5.0^a^	−7.4	−2.4	− 5.4^a^	−9.1	− 1.6	6.2	−2.0	15.1	− 1.0	−5.1	3.3
Poland	2007–2017	0.4	−0.5	1.2	1.3	−0.5	3.2	0.1	− 1.9	2.1	−2.5	−11	6.9	4.7	−1.4	11.2
Portugal	2011–2017	−1.4^a^	−2.3	−0.6	16.0^a^	12	20.2	−10.4^a^	−17.9	−2.2	−9.2	−27.7	14.0	−6.1	− 16.9	6.1
Slovenia	2011–2017	0.0	−0.1	0.2	−1.7	− 12.9	11.0	5.0	−6.1	17.4	17.1^a^	5.7	29.6	−3.0	−15.6	11.4
Spain	2004–2017	1.0^a^	0.3	1.6	−1.1	−3.7	1.7	−1.7	−4.2	0.9	6.8	−2.3	16.8	1.0	−4.8	7.2
Sweden	2004–2017	−0.4	−1.0	0.2	2.7^a^	1.4	3.9	5.3^a^	1.1	9.7	1.5	−9.2	13.4	−4.2	−11.5	3.7
Switzerland	2004–2017	−0.1	−0.9	0.8	2.3^a^	0.5	4.1	6.9^a^	4.2	9.7	3.5	−11.1	20.4	−2.7	−7.4	2.2
**Women**																
Austria	2004–2017	0.6^a^	0.0	1.2	3.5^a^	1.4	5.6	1.2	−2.5	5.1	6	−2.8	15.6	0.1	−6.1	6.7
Belgium	2004–2017	−0.2	− 0.8	0.5	1.7^a^	0.6	2.8	3.5^a^	1.9	5.2	4.6	−3.2	13.1	−0.5	−4.8	4.1
Czech Republic	2007–2017	0.5	−0.1	1.1	0.8	−2.9	4.6	6.0^a^	1.4	10.9	4.2	−5.1	14.5	−0.7	−4.8	3.6
Denmark	2004–2017	0.5	−0.4	1.4	0.8	−1.1	2.8	−0.5	− 1.3	0.3	1.6	−7.4	11.4	−4.7	−11.3	2.3
Estonia	2011–2017	−1.6	−3.4	0.2	−7.0	−20.2	8.5	−3.0	−14.6	10.2	7.0^a^	1.0	13.3	−4.7	−18.5	11.6
France	2004–2017	− 0.5	−1.1	0.1	0.3	−2.4	3.1	2.2^a^	0.8	3.6	−0.3	−6.6	6.5	−0.8	−3.2	1.7
Germany	2004–2017	0.8^a^	0.1	1.5	3.8^a^	0.4	7.3	8.5^a^	5.8	11.3	0.3	−6.6	7.8	1.8	−3.0	7.0
Netherlands	2004–2017	0.4	−1.4	2.2	3.7	−1.3	9.0	4.9	−6.5	17.7	−0.4	−12.9	14.0	0.9	−10.9	14.1
Italy	2004–2017	−0.3	− 0.9	0.3	− 5.2^a^	− 7.7	−2.7	− 5.4^a^	−9.0	−1.6	5.7^a^	0.0	11.7	−1.1	− 5.1	3.1
Poland	2007–2017	0.3	− 0.6	1.2	1.0	−0.8	2.8	−0.1	−1.9	1.8	−1.9	−7.2	3.6	4.6	−1.5	11
Portugal	2011–2017	−1.3^a^	−2.2	− 0.4	15.8^a^	12.1	19.6	−10.4^a^	−18	−2.0	− 9.0	− 28	15.2	−6.3	− 16.7	5.4
Slovenia	2011–2017	0.1	−0.2	0.4	−1.8	−12.9	10.6	4.6	−6.4	17.0	17.1^a^	3.8	32.0	−3.2	−15.7	11.1
Spain	2004–2017	0.9^a^	0.4	1.4	−1.4	− 4.0	1.3	−1.7	− 4.1	0.7	6.3^a^	0.0	13.0	0.9	−5.0	7.1
Sweden	2004–2017	− 0.4	−1.0	0.2	2.3^a^	1.1	3.5	5.1^a^	1.0	9.4	1.1	−6.8	9.7	−4.3	−11.5	3.5
Switzerland	2004–2017	−0.1	−0.8	0.7	2.0^a^	0.2	3.7	6.7^a^	4.0	9.5	3.1	−8.8	16.6	−2.8	−7.6	2.2

^a^Statistically significant (*p* < 0.05); *CM* Cardiometabolic diseases, *RE* Respiratory diseases, *MS* Musculoskeletal diseases, *ND* Neurodegenerative diseases, *CA* Cancer

## Discussion

Our results show the prevalence trends of multimorbidity community-dwelling adults aged 50 and over, living in 15 European countries. The observed data reveal an increase in the prevalence of multimorbidity in the countries of central Europe (Austria, Belgium, Czech Republic, France, Germany and Switzerland) and Spain in men and women, and in the Netherlands in men. Stability was observed in the analysis by periods in some southern (Portugal and Italy), northern (Denmark and Sweden) and eastern (Estonia, Poland and Slovenia) European countries. In the case of Portugal, Estonia, Poland and Slovenia, the short period limits the results of the analysis. However, in some of these countries it was possible to identify changes when analysing age groups and prevalence by disease group.

The prevalence of multimorbidity was higher in the Czech Republic, Poland and Portugal, whereas the countries with the lowest multimorbidity prevalence were Switzerland, Sweden and the Netherlands. Similar differences were found by a study using SHARE data from wave 5 [15]. However, it is necessary to make some methodological considerations when comparing data with other studies. Firstly, the definition of multimorbidity is heterogeneous, which makes it difficult to compare prevalence data [16, 17]. The presence of two or more chronic conditions is the most common definition [18], but the number of included non-communicable diseases varies between 4 and 102 in the literature [19]. Therefore, the changes in prevalence may occur as a result of diseases that were included in the composition of the multimorbidity definition in a time series study. Another aspect that should be considered is the method used to collect the information. Some studies have used clinical records in hospitals or primary care units, while others collected information through self-report [14, 20–22]. Self-report is the most feasible method for population-based epidemiological studies, but it has the potential to underestimate the prevalence [19].

A study conducted in Nijmegen, the Netherlands, analysed the prevalence trend of multimorbidity for the period 1985 to 2005 through a morbidity register of 13,584 primary care patients, without an age limit. The proportion of patients without chronic diseases decreased from 70 to 63%, and the proportion of individuals with one chronic disease was stable while those with two increased from 6.7 to 8% [23]. Two other studies also carried out in the Netherlands found an increase in the prevalence of multimorbidity. Tacken et al. [7] included diabetes mellitus as well as cardiovascular and respiratory diseases, and found an increase in prevalence during the period 2003 to 2009. As did Oostrom et al., who considered the co-occurrence of two or more of 28 diseases during the period 2001–2011 [6].

Prevalence of multimorbidity has also been increasing in non-European countries. A trend analysis was realised in Hong Kong with 69,636 adults aged 35 or over who participated in the surveys in 1999, 2001, 2005 and 2008. Multimorbidity was defined as presenting two or more chronic conditions from a list of 14 [8]. Further, a study conducted in Canada with data of 1996–97, 2005 and 2012–13 identified an increase in the prevalence of multimorbidity that may be attributed to an increase of obesity [24]. A pooled analysis of individuals from cohort studies from the USA and Europe shows that the risk of cardiometabolic multimorbidity increases as BMI increases [25].

Overall, the increase or reduction in the prevalence of multimorbidity could be explained by two circumstances: changes in the prevalence of the main risk factors, such as tobacco and alcohol consumption, diet or practice of physical activity; or changes in the classification system and/or improvement in diagnosis. Regarding the first situation, a study that also analysed data from the SHARE project identified a non-significant increase of obesity and overweight in France, Switzerland and Denmark. On the other hand, a significant decrease in the prevalence of overweight was observed for Spain (− 3.5, 95% CI: − 6.1 to − 0.9) and Italy (− 4.3, 95% CI: − 7, 3 to − 1.3). For obesity, there was a significant increase in Germany (5.8, 95% CI, 1.8–9.9) and a significant reduction in Spain (− 4.7, 95% CI: − 8.8 to − 0.5) [26] These results provide clues that could contribute to explaining the differences in the multimorbidity trends that we find by countries, but not the increase in Spain, and the changes in musculoskeletal diseases trends, which has obesity as a risk factor.

The analysis by disease group showed more changes in the prevalence of multimorbidity attributed to cardiometabolic, respiratory and musculoskeletal diseases. The prevalence of cardiometabolic diseases is highest among those with multimorbidity, with a significant reduction only in Portugal. In a global study analysing trends of prevalence of diseases and years lived with disability (YLDs) by major cause groups, the main burden was attributed to musculoskeletal, mental, neurological and respiratory conditions [1].

The prevalence of neurodegenerative diseases increased in four countries analysed. Neurodegenerative diseases in men increased in Estonia and Slovenia. In women, the increase was observed in Estonia, Slovenia, Italy and Spain. A longitudinal study on Alzheimer’s disease verified an increase in neurodegenerative diseases in most European countries from 1994 to 2013. This study analysed mortality trends, identifying an increasing trend in both sexes in all countries except Germany and Malta, where a reduction was observed. The authors presented two main hypotheses for this finding: better diagnoses attributed to the implementation of ICD-10; and the contribution of environmental factors [27].

Musculoskeletal diseases presented an increase in northern and central countries (Belgium, Czech Republic, France, Germany, Sweden and Switzerland) and a reduction in Italy and Portugal. As observed in our study, different trends of prevalence of osteoarthritis or rheumatoid arthritis can be found in the literature [28]. A study conducted in the United States described a reduction in the prevalence of rheumatoid arthritis and an increase for osteoarthritis [29], and another identified stability for rheumatoid arthritis in Canada [30]. These variations are probably related to risk factors such as obesity and genetic factors [31].

Several studies show that the prevalence of multimorbidity increases with age [21, 32, 33]. Stratified analysis for 10-year age groups indicates in our results a possible cohort effect in some countries, such as the reduction among men and women in Italy for the younger age groups (50–59 and 60–69) and increase in men in the 80 and over age group. Furthermore, the increase in multimorbidity occurred in all age groups in men in Austria, Czech Republic and Spain, and in women in Germany. Chatterji et al., analysing SHARE data, did not find any cohort effect when analysing limitations in the activities of daily living, a strong indicator of multimorbidity. However, the study analysed the pooled cohort effect and no stratification by country was applied [34].

Concerning the limitations of our study, we emphasise that this is an ecological study, and therefore it is not possible to establish a causal relationship between the exposure to risk factors and the prevalence of each disease. Another aspect to consider is the sampling of the oldest age group in the SHARE project, which may affect comparability across countries among those aged 80 and over [35]. Similar to other studies, we decided to include this age group due to its relevance to the issue. Another limitation is self-reported information and sociocultural differences between countries (e.g. language). However, these difficulties have been compensated by the harmonisation and standardisation of the questionnaires and data collection procedures, guaranteed by the meticulous process of cultural adaptation, as well as by the high professionalism of highly trained researchers and interviewers.

Regarding the strengths of our work, we highlight that this study on multimorbidity prevalence trends included the largest number of countries with detailed analysis by age and disease groups. Additionally, one advantage of a longitudinal study such as SHARE is the quality and quantity of data of different waves and countries due to the standardisation of data collection.

## Conclusions

The information provided in this paper can be useful for planning health services and prevention policies addressing modifiable risk factors in the population. Our results showed a large variability in the prevalence of multimorbidity in adults aged 50 and over between European countries. An upward trend was identified in some countries of central Europe and Spain in both sexes, and in the Netherlands among men. On the other hand, stability was observed in some southern (Portugal, and Italy) and eastern European countries (Estonia, Poland and Slovenia).

## Data Availability

The source of raw data analysed in this study is free available upon request through the website: http://www.share-project.org/data-access.html. The specific datasets used and analysed during the current study are available from the corresponding author on reasonable request.

## Abbreviations

APC: Annual Percentage Change
CESS: Centre for Health and Social Care Research
CI: Confidence Interval
M_3_O: Research group on Methodology, Methods, Models and Outcomes of Health and Social Sciences
UVic-UCC: University of Vic-Central University of Catalonia
SHARE: Survey of Health, Ageing and Retirement in Europe
YLD: Years Lived with Disability
